# The impact of medical tourism on colorectal screening among Korean Americans: A community-based cross-sectional study

**DOI:** 10.1186/s12885-016-2965-y

**Published:** 2016-12-01

**Authors:** Linda K. Ko, Victoria M. Taylor, Jihye Yoon, Wade K. Copeland, Joo Ha Hwang, Eun Jeong Lee, John Inadomi

**Affiliations:** 1 Division of Public Health Sciences, Fred Hutchinson Cancer Research Center, Department of Health Services, University of Washington School of Public Health, 1100 Fairview Ave. N, M3-B232, Seattle, WA 98109-1024 USA; 2 Division of Public Health Sciences, Fred Hutchinson Cancer Research Center, Seattle, WA USA; 3 Division of Gastroenterology, University of Washington School of Medicine, Seattle, WA USA; 4 National Asian Pacific Center on Aging, Seattle, WA USA

**Keywords:** Colorectal cancer screening, Medical tourism, Korean Americans

## Abstract

**Background:**

Colorectal cancer (CRC) remains the most commonly diagnosed cancer among Korean Americans (KAs) in part due to low screening rates. Recent studies suggest that some KA patients engage in medical tourism and receive medical care in their home country. The impact of medical tourism on CRC screening is unknown. The purpose of this paper was to 1) investigate the frequency of medical tourism, 2) examine the association between medical tourism and CRC screening, and 3) characterize KA patients who engage in medical tourism.

**Methods:**

This is a community-based, cross-sectional study involving self-administered questionnaires conducted from August 2013 to October 2013. Data was collected on 193 KA patients, ages 50–75, residing in the Seattle metropolitan area. The outcome variable is up-to-date with CRC screening, defined as having had a stool test (Fecal Occult Blood Test or Fecal Immunochemical Test) within the past year or a colonoscopy within 10 years. Predictor variables are socio-demographics, health factors, acculturation, knowledge, financial concerns for medical care costs, and medical tourism.

**Results:**

In multi-variate modeling, medical tourism was significantly related to being up-to-date with CRC screening. Participants who engaged in medical tourism had 8.91 (95% CI: 3.89–23.89) greater odds of being up-to-date with CRC screening compared to those who did not travel for healthcare. Factors associated with engaging in medical tourism were lack of insurance coverage (*P* = 0.008), higher levels of education (*P* = 0.003), not having a usual place of care (*P* = 0.002), older age at immigration (*P* = 0.009), shorter years-of-stay in the US (*P* = 0.003), and being less likely to speak English well (*P* = 0.03).

**Conclusions:**

This study identifies the impact of medical tourism on CRC screening and characteristics of KA patients who report engaging in medical tourism. Healthcare providers in the US should be aware of the customary nature of medical tourism among KAs and consider assessing medical tests done abroad when providing cancer care.

**Trial registration:**

Not applicable.

## Background

The US Preventive Services Task Force recommends screening for colorectal cancer (CRC) using stool testing (Fecal Occult Blood Test or Fecal Immunochemical Test) annually, flexible sigmoidoscopy every 5 years with stool test every 3 years, and colonoscopy every 10 years, beginning at age 50 and continuing until age 75 [1]. Over the last two decades, there has been a steady increase in the use of CRC screening in the United States [2]. Prevention and early detection of CRC through the use of screening tests have resulted in better prognosis and longer survival and reduced disease incidence and mortality [1–3]. The increase in CRC screening use, however, has not occurred evenly across the US population, and minority and immigrant populations still disproportionately underutilize CRC screening. One such minority and immigrant population is Korean Americans (KAs) [4]. CRC incidence rates have steadily increased among KAs since 1990 [2, 4, 5]. Over the last 15 years, CRC has been the most commonly diagnosed cancer among KAs, [2, 4, 5] and CRC screening use is low in this population [4–7].

Research shows that KAs who do not undergo a CRC screening test are largely uninsured, often recent immigrants, and frequently have limited English proficiency [6–8]. These factors position them to be disconnected from the US healthcare system and the larger public health efforts directed at promoting CRC screening among underserved populations. In addition, some KAs seek healthcare from KA doctors who speak Korean; many of these professionals received their training in South Korea and may not adhere to the CRC screening guidelines in the US [9, 10].

A recent body of evidence suggests that some KAs may be engaging in medical tourism and traveling to their home country to receive preventive care including cancer screening [11]. For example, a study using qualitative methodology with focus groups found that some KA women reported traveling to their home country to receive screening for breast and cervical cancer [11]. The Centers for Disease Control and Prevention have reported that US patients who engage in medical tourism are largely immigrants who return to their home country for care [12]. A recent report from the Korea International Medical Association noted that medical tourism has increased as a result of the foreign patient legislation law in 2009 [13]. This law enables foreign patients and their families to obtain longer-term medical visas and authorizes local hospitals to market medical tourism to foreign patients [13]. As a result, medical tourism has emerged as a vibrant industry, establishing South Korea as a host country for providing quality, affordable healthcare services to KAs [14].

An understanding of medical tourism is important because it could potentially have an impact on KA patients’ preventive care, follow-up, and treatment for CRC as well as other medical conditions. In South Korea, although a stool test (Fecal Occult Blood Test or Fecal Immunochemical Test) is usually the first choice of recommendation among South Korean providers, colonoscopy is readily available for those who can pay out of pocket and is done every 5 years [15]. In addition, the cost of receiving a colonoscopy in South Korea is approximately 2.3% of the cost of colonoscopy in the US; this lower cost may encourage individuals to get screened for CRC as it somewhat removes a financial barrier [16]. However, we know little about the occurrence of medical tourism among KAs or the extent to which medical tourism is associated with getting a CRC screening test among KAs. Additionally, no previous studies have characterized the patients who choose to engage in medical tourism or identified factors that play a role in KAs’ decision to engage in medical tourism. The purpose of this study is to examine the topic of medical tourism and CRC screening including 1) the frequency of medical tourism, 2) the association between medical tourism and CRC screening in relation to established CRC screening predictors, and 3) characteristics of KA patients who engage in medical tourism.

## Methods

### Study Overview

This cross-sectional, observational study was conducted in the Seattle metropolitan area of Washington State (WA). In WA, Asians are the second largest minority population and KAs are the fourth largest Asian group; most WA KAs reside in the Seattle metropolitan area [17]. The survey was administered over a 3-month period from August 2013 to October 2013. Our three data collectors were all bilingual KA women. Participants completed an in-person, 10–15 min, self-administered paper-and-pen survey in their preferred language (Korean or English). All the participants chose to complete their survey in Korean. Participants received a gift bag with health promotion materials as a token of appreciation for their time. Study materials were translated from English into Korean using standard forward translation methods. This study was approved by the Institutional Review Board and the Ethics Committee of the Fred Hutchinson Cancer Research Center (IRB # 8051). Verbal consent was obtained from all the study participants prior to participation. An advisory group of KA community leaders provided guidance regarding participant recruitment, survey procedures, and survey instruments. Our study had a total sample size of 193.

### Study Participants

Potential survey participants were identified for this study at Korean community health events, Korean American churches, and community-based organizations that serve Korean Americans. Participants’ inclusion criteria included being 50–75 years of age, residing in the Seattle metropolitan area, and self-identifying as “Korean” or “Korean American.” Of the 382 participants that we approached, 79 participants (21%) were not age eligible (younger than 50 or older than 75). Of the 303 remaining participants, 193 agree to participate in the survey, giving us a 64% response rate.

### Survey Instruments

Our survey questions were adapted from the National Health Interview Survey [18] and studies on CRC screening studies among Korean Americans [6, 7], medical tourism [19], and financial concerns for medical costs [20].

*Outcome Variable*: The outcome variable was up-to-date with CRC screening, which was defined as having an annual stool test with Fecal Occult Blood Test or Fecal Immunochemical Test or colonoscopy every 10 years [1]. Participants were not queried about sigmoidoscopy as recommendation of this CRC screening test by physicians in the Seattle metropolitan area is rare.

*Predictor variables*: *Socio-demographic characteristics* included age, gender, employment, marital status, health insurance, education level, and household income. *Health factors* were measured with four questions: current health status, number of chronic disease diagnoses (diabetes, heart disease, high blood pressure, arthritis, hepatitis, and high cholesterol), family history of CRC, and having a usual place for medical care in the US. *Acculturation* was measured with three questions: age of immigration, years living in the US, and English speaking proficiency. *Knowledge of CRC screening test* was measured with two questions: knowledge about when people should begin testing for CRC and whether participants agreed or disagreed with a statement that there was only one CRC screening option. *Medical tourism* included three questions: number of times individuals have traveled outside of the US to receive healthcare within the past 5 years, the most recent date of travel, and the country of travel. *Worries about costs of care* were measured with two questions: *How worried are you right now about not being able to pay medical costs for general healthcare? How worried are you right now about not being able to pay for a serious illnesses or an accident*? Costs for general healthcare included annual providers’ visits and routine medical tests. Costs for serious illnesses included surgeries, medical treatments for diabetes, cancer and other diseases as well as injuries due to accidents.

### Data Analysis

All analyses were done in SAS Version 9.4. and R. Descriptive analyses generated frequencies for categorical variables and means for continuous variables. Bivariate analyses were conducted with logistic regressions. Multi-variate analyses were conducted using logistic regression techniques to identify predictors that were significantly associated with being up-to-date with CRC screening. Multi-variate analysis included variables that were shown to be associated with a CRC screening test in other studies with KAs; we have also included variables that were significant at 0.05 level. Some predictors that were potentially related were not included in multi-variate analyses due to concerns about collinearity. Significance level was set at *P* < 0.05 and was unadjusted for multiple comparisons.

## Results

### Participant Characteristics

Participants’ characteristics are shown in Table 1. The mean age was 62 years old (±7.19). More than half of the KAs were female (63%), married (79%), not insured (59%) and had some college education (52%). About half were employed (50%) and had an income greater than $20,000 (51%). Many reported good health (52%) and had a place of usual care in the US (63%). Participants reported an average of 2 (±1.70) diagnoses of chronic diseases, and the majority did not have a family history of CRC (79%). The mean age of immigration to the US was 39 years old (±11.44), average years living in the US was 23 years (±10.52), and most did not speak English well (72%); all participants reported being born outside of the US. The majority reported incorrect knowledge on when an individual should begin testing for CRC screening (75%) and the number of CRC screening tests available (83%). Fifty-seven percent of the participants reported being up-to-date with a CRC screening test, with 2% reporting having a stool test and 98% reporting colonoscopy. About half reported being worried about medical costs for general care (51%) and being worried about medical costs for serious illness (62%). One third of the patients (33%) reported traveling outside of the US for medical care, on average 2.5 times within the past 5 years, and the most common destination was South Korea (95%).

**Table 1:** Bivariate Relationship Between Predictors and CRC Screening (*n* = 193)

	Sample *n* (%)	Not Screened *n* (%)	Screened *n* (%)	*P* value
Socio-Demographics				
Age in years; Mean (SD)^a^	61.59 (7.19)	59.91 (7.12)	62.57 (6.88)	0.01
Gender				
Male	70 (37)	33 (47)	37 (53)	0.41
Female	117 (63)	48 (41)	69 (59)	
Employment status				
Working full time	30 (16)	15 (50)	15 (50)	0.32
Working part time	19 (10)	11 (58)	8 (42)	
Self-employed	45 (24)	19 (42)	26 (58)	
Unemployed	91 (49)	34 (37)	57 (63)	
Marital status				
Married	145 (79)	63 (43)	82 (57)	0.99
Unmarried	39 (21)	17 (44)	22 (56)	
Health insurance				
Insured	73 (41)	26 (36)	47 (64)	0.06
Not insured	105 (59)	52 (50)	53 (50)	
Education status				
Less than high school	31 (17)	14 (45)	17 (55)	0.25
High school graduate or GED^b^	57 (31)	25 (44)	32 (56)	
Some college	21 (11)	13 (62)	8 (38)	
College graduate and more	75 (41)	28 (37)	47 (63)	
Annual household income				
Less than $20,000	62 (35)	29 (47)	33 (53)	0.73
$20,000 up to $39,999	32 (18)	13 (41)	19 (59)	
$40,000 up to $59,999	33 (19)	14 (42)	19 (58)	
$60,000 and more	25 (14)	9 (36)	16 (64)	
Don’t know	26 (15)	14 (54)	12 (46)	
Health Factors				
Current health				
Good or excellent	96 (52)	37 (39)	59 (61)	0.16
Fair or poor	88 (48)	43 (49)	45 (51)	
Number of diagnoses				
0	34 (18)	20 (59)	14 (41)	0.04
1 and more	153 (82)	61 (40)	92 (60)	
Family history of CRC^c^				
Yes	31 (17)	10 (32)	21 (68)	0.35
No	148 (79)	68 (46)	80 (54)	
Don’t know	8 (4)	3 (38)	5 (62)	
Place of usual care in US				
Yes	116 (63)	44 (38)	72 (62)	0.06
No	67 (37)	35 (52)	32 (48)	
Acculturation				
Age immigrated to the US; Mean (SD)	38.83 (11.44)	37.48 (11.04)	39.76 (11.46)	0.17
Years lived in the US; Mean (SD)	22.72 (10.52)	22.27 (10.1)	22.7 (10.79)	0.78
English speaking proficiency				
Well	50 (28)	21 (41)	30 (59)	0.71
Not well	129 (72)	57 (44)	72 (56)	
CRC Screening Knowledge				
Age screening begins				
Correct	46 (25)	16 (35)	30 (65)	0.15
Incorrect	139 (75)	65 (47)	74 (53)	
Only one test available				
Correct	31 (17)	12 (39)	19 (61)	0.54
Incorrect	152 (83)	68 (45)	84 (55)	
Medical Cost				
Worries about medical costs for general care				
Worried	93 (51)	48 (52)	45 (48)	0.03
Not worried	88 (49)	31 (35)	57 (65)	
Worries about medical costs for serious illness				
Worried	113 (62)	56 (50)	57 (50)	0.03
Not worried	70 (38)	23 (33)	47 (67)	
Medical Tourism				
Number of times traveled outside of the US				
0	121 (67)	67 (55)	54 (45)	<0.001
1 and more	60 (33)	11 (18)	49 (82)	
When last traveled				
A year ago or less	23 (37)	2 (9)	21 (91)	0.12
1-3 years ago	25 (40)	8 (32)	17 (68)	
3+ years ago	15 (24)	3 (20)	12 (80)	
Country traveled^d^				
Korea	60 (95)	11 (18)	49 (82)	0.08
Not Korea	3 (5)	2 (67)	1 (33)	

^a^SD = Standard Deviation

^b^GED = General Educational Development

^c^CRC = Colorectal Cancer

^d^Only those who report traveling outside of the country for medical care. Bivariate analysis using logistic regressions

### Bivariate Relationship between Socio-demographics, Health Factors, Acculturation, Knowledge, Medical Costs, and Medical Tourism and CRC Screening

There was a significant relationship between age, having a diagnosis of chronic illness, worries about medical care costs, and having traveled outside of the country for medical care and being up-to-date with CRC screening (Table 1). Older participants were more likely to be screened for CRC compared to the younger participants (62.57 ± 7.12 vs. 59.91 ± 6.88; *P* = 0.01). Participants who reported one or more diagnoses of chronic illness were more likely to be up-to-date with CRC screening compared to those reporting no diagnosis (60 vs. 41%; *P* = 0.04). Additionally, participants who reported being not worried about general medical care costs (Not worried: 65% vs. Worried: 48%; *P* = 0.03) or about costs for serious illness (Not worried: 67% vs. Worried: 50%; *P* = 0.03) were more likely to be screened for CRC compared to those who reported being worried. Finally, participants who had traveled outside of the country for medical care once or more were more likely to be up-to-date with CRC screening compared to those who reported no travel (82 vs. 45%; *P* < 0.001). Health insurance status and having a usual place of care showed a trend towards significance; participants who were insured (64 vs. 50%; *P* = 0.06) and had a usual place of care (62 vs. 48%; *P* = 0.06) were more likely to be up-to-date with CRC screening compared to those who were uninsured and did not have a usual place for care.

### Multi-variate Relationship Between Predictors and CRC Screening

Medical tourism was the only variable that was significantly associated with CRC screening (Table 2); participants who reported traveling outside of the country for medical care had 8.91 (95% CI: 3.85–23.89) higher odds of being up-to-date with CRC screening compared to those who did not travel. None of the other variables, including age, age of immigration to the US, knowledge about when CRC screening should begin, family history, health insurance status, having a place of usual care, having a diagnosis of a chronic illness, and worries about general medical costs were statistically associated with being-up-to-date with a CRC screening test.

**Table 2:** Multi-variate Relationship Between Predictors and CRC Screening

	OR^a^ (95% CI)	*P* value
Intercept	0.43 (0.01, 30.36)	0.69
Age	1.03 (0.96, 1.11)	0.35
Health insurance		
Insured	1	
Not insured	0.71 (0.25, 2.06)	0.53
Age immigrated to the US	0.99 (0.95, 1.03)	0.54
Age screening begins		
Correct	1	
Not correct	0.53 (0.22, 1.21)	0.14
Number of diagnoses		
0	1	
1 and more	1.97 (0.77, 5.26)	0.16
Family history of CRC^b^		
Yes	1	
No	0.51 (0.17, 1.4)	0.20
Don’t know	0.82 (0.10, 9.18)	0.86
Place of usual care in US		
Yes	1	
No	0.44 (0.16, 1.14)	0.10
Worries about medical costs for general care		
Not worried	1	
Worried	0.75 (0.34, 1.68)	0.49
Number of times traveled outside of the US		
0	1	
1 and more	8.91(3.85, 23.89)	<0.001

^a^OR = Odds Ratio

^b^CRC = Colorectal Cancer. Multi-variate analysis using logistic regressions between the predictors and being up-to-date with CRC screening

### Bivariate Relationship Between Socio-demographics, Health Factors, Acculturation, Knowledge, and Medical Costs and Medical Tourism

There was a significant relationship between health insurance status, education level, having a usual place for care, age of immigration to the US, years-of-stay in the US, and English proficiency and medical tourism (Table 3). Participants who reported traveling outside of the country for medical care were less likely to be insured (23 vs. 77%; *P* = 0.008) and to report having a usual place for care (25 vs. 75%; *P* = 0.002). They were also less acculturated compared to those who did not travel, having immigrated to the US at an older age (37.40 ± 11.35 vs. 42.13 ± 11.36; *P* = 0.009), having shorter years-of-stay in the US (24.37 ± 10.63 vs. 19.33 ± 9.86; *P* = 0.003), and being less likely to speak English well (22 vs. 78%; *P* = 0.03).

**Table 3:** Bivariate Relationship Between Socio-demographics, Health Factors, Acculturation, Knowledge, and Medical Costs and Medical Tourism

		Medical Tourism		
	Sample *n* (%)	No *n* (%)	Once or More *n* (%)	*P* value
Socio-Demographics				
Age in years; Mean (SD)^a^	61.59 (7.19)	61.82 (7.52)	61.27 (6.5)	0.62
Gender				
Male	69 (37)	47 (68)	22 (32)	0.96
Female	118 (63)	80 (68)	38 (32)	
Employment status				
Working full time	30 (16)	23 (77)	7 (23)	0.25
Working part time	17 (9)	13 (76)	4 (24)	
Self-employed	44 (24)	25 (57)	19 (43)	
Unemployed	94 (51)	64 (68)	30 (32)	
Marital status				
Married	144 (78)	94 (65)	50 (35)	0.24
Unmarried	40 (22)	30 (75)	10 (25)	
Health insurance				
Insured	75 (42)	58 (77)	17 (23)	0.008
Not insured	104 (58)	61 (59)	43 (41)	
Education status				
Less than high school	30 (16)	27 (90)	3 (10)	0.003
High school graduate or GED^b^	57 (31)	36 (63)	21 (37)	
Some college	21 (12)	17 (81)	4 (19)	
College graduate and more	74 (41)	43 (58)	31 (42)	
Annual household income				
Less than $20,000	64 (36)	42 (66)	22 (34)	0.40
$20,000 up to $39,999	30 (17)	18 (60)	12 (40)	
$40,000 up to $59,999	31 (17)	22 (71)	9 (29)	
$60,000+	25 (14)	14 (56)	11 (44)	
Don’t know	28 (16)	22 (79)	6 (21)	
Health Factors				
Current health				
Good or excellent	97 (53)	66 (68)	31 (32)	0.84
Fair or poor	87 (47)	58 (67)	29 (33)	
Number of diagnoses				
0	35 (19)	27 (77)	8 (23)	0.18
1 and more	152 (81)	100 (66)	52 (34)	
Family history of CRC^c^				
Yes	32 (17)	19 (59)	13 (41)	0.46
No	146 (78)	101 (69)	45 (31)	
Don’t know	9 (5)	7 (78)	2 (22)	
Place of usual care in US				
Yes	117 (64)	88 (75)	29 (25)	0.002
No	66 (36)	35 (53)	31 (47)	
Acculturation				
Age immigrated to the US; Mean (SD)	38.83 (11.44)	37.4 (11.35)	42.13 (11.36)	0.01
Years lived in the US; Mean (SD)	22.72 (10.52)	24.37 (10.63)	19.33 (9.86)	0.003
English speaking proficiency				
Well	51 (28)	40 (78)	11 (22)	0.03
Not well	129 (72)	80 (62)	49 (38)	
CRC Screening Knowledge				
Age screening begins				
Correct	47 (25)	32 (68)	15 (32)	0.95
Not correct	139 (75)	94 (68)	45 (32)	
Only one test available				
Correct	31 (17)	22 (71)	9 (29)	0.64
Not correct	153 (83)	102 (67)	51 (33)	
Medical Cost				
Worries about medical costs for general care				
Worried	92 (51)	59 (64)	33 (36)	0.43
Not worried	89 (49)	62 (70)	27 (30)	
Worries about medical costs for serious illness				
Worried	114 (62)	72 (63)	42 (37)	0.12
Not worried	70 (38)	52 (74)	18 (26)	

^a^SD = Standard Deviation

^b^GED = General Education Development

^c^CRC = Colorectal Cancer. Bivariate analysis using logistic regressions

## Discussion

This study examined the frequency of medical tourism among KA patients, the relative predictability of medical tourism on CRC screening, and characteristics of KA patients who engage in medical tourism. Our findings show that one third (33%) of the participants who we surveyed reported traveling to South Korea for medical care. Medical tourism emerged as the strongest predictor of CRC screening in multi-variate analysis, while other established predictors such as age, education, acculturation, health insurance status, and having a usual place of care were not statistically significant.

Our results show that the majority (77%) of KA patients who engage in medical tourism had traveled within the last 3 years. A previous report noted a steady increase of US patients engaging in medical tourism, mainly KAs, since 2009, when South Korea passed the foreign patient legislation law [21, 22]. This rise has been attributed to an aggressive joint marketing strategy by the South Korean government and private sectors targeting KAs [13, 14, 23]. For instance, a large Korean hospital has established a local office in Los Angeles, where many KAs reside [24, 25]; other South Korean hospitals have partnered with Korean travel agencies in the US to promote medical tourism as part of vacation packages [25, 26]. These strategies, coupled with word of mouth from those who had a positive experience, may have helped spread the information [11]. Findings from a qualitative research study among KA women showed that KA women who had engaged in medical tourism or were contemplating medical tourism viewed it as offering multiple benefits, including relatively low costs, convenience, and having access to good quality medical care and advanced technologies as well as opportunities to visit their homeland and to enjoy vacations out of the US [11]. Patients’ perceived benefits appear to outweigh the challenges or risks associated with seeking preventive care in South Korea, such as delayed healthcare, flying long hours, and expensive travel and accommodations costs abroad [11].

Engaging in medical tourism was the strongest predictor of CRC screening among KAs. All the other established predictors of CRC screening were not significantly associated with CRC screening in the multi-variate analysis. At first glance, this finding may seem contrary to the current literature. However, a closer look may provide insights into the changing global medical landscape and the impact of this contextual factor in KAs’ cancer screening behaviors. For instance, most of the findings from prior studies on CRC screening among KAs report data gathered before 2009, when medical tourism was rare [13]. Additionally, a recent report indicates that the sharpest increase in medical tourism among US patients occurred between 2011 and 2012 [27]. As our study was conducted in 2013, we may have captured not only the effect of the legislation, but also the timeframe when the legislation had the largest impact. Nevertheless, CRC screening rates among KAs remain very low and have always been low. Since no other previous studies have assessed medical tourism, we do not know whether KAs who were screened for CRC before the 2009 legislation were screened outside of the US. The low screening rates may be a reflection of delays with preventive care associated with medical tourism.

It is important to note that patients who reported medical tourism also reported lower levels of acculturation, less insurance coverage, and not having a usual place for medical care. Furthermore, patients who engaged in medical tourism reported older age of immigration, shorter years-of-stay in the US, and were less likely to speak English well compared to those who did not report medical tourism. These patients may have been more familiar with the medical system in South Korea than in the US, facilitating their decision to seek care outside of the US. Regardless of medical tourism, more than half of the patients reported working, whether full time, part time, or self-employed; however, only 23% of the patients who engaged in medical tourism were insured, compared to 77% who did not engage in medical tourism. This finding may be indicative of their scarce employment options, given the older age of immigration and English language proficiency, and their limited access to employer-sponsored health insurance plans.

Most Korean hospitals provide streamlined integrated preventive care, including cancer screening, without the need for referrals and multiple visits to different providers and offices. Research shows that KA patients in the US choose to receive care in South Korea for procedures that involve high out-of-pocket costs such as co-pays and deductibles [11]. As shown in the current study as well as other studies, KA patients tend to prefer colonoscopy over stool testing, and believe that colonoscopy is the only test available for CRC [28]. KA patients’ preference for colonoscopy may explain the high rates of medical tourism among the uninsured (68%), but also the insured (28%).

Providers in the US need to be aware that KA patients may follow the CRC screening guidelines from South Korea and this country’s medical care system. In South Korea, although a stool test is usually the first choice of recommendation among South Korean providers, colonoscopy is readily available for those who can pay out of pocket. The cost of colonoscopy in South Korea is approximately 2.3% of the cost of colonoscopy in the US (approximately $130 to $200 US dollars) [16]. Mistrust could arise if KA patients’ beliefs about CRC care are different from US providers’ recommendations. Providers should take the time to discuss US CRC guidelines and acknowledge the differences between the US and South Korean guidelines.

This study had several limitations. First, the data comes from a convenience sample. Therefore, we cannot generalize the findings cannot be generalized to all KAs in metropolitan Seattle or KAs in other geographic regions, but are limited to KA participants in our study. However, convenience sampling is often used in immigrant populations that tend to be clustered in specific geographic areas. Second, all of the responses were self-reported. We could not confirm CRC screening with electronic medical records or confirm frequency of medical tourism with travel boarding passes. However, other studies of CRC screening have used self-report [29, 30], and our findings with respect to participants’ medical tourism are similar to those of prior research [11]. Finally, the measure of medical tourism may have served as a proxy for other variables such as income and insurance.

## Conclusions

The results of our study indicate that the global medical landscape is changing, and these changes are impacting KAs’ CRC screening behaviors. It is important for providers to assess medical tourism during routine clinic visits or when patients show delays in preventive cancer care or treatment. This step is critical for cancer care, particularly in regard to adherence to screening guidelines, follow-up from abnormal results in South Korea, and timely adherence to treatment recommendations.

## Abbreviations

CRC: Colorectal cancer screening
GED: General Educational Development
KA: Korean American
OR: Odds ratio
SD: Standard deviation
US: United States of America
WA: Washington
